# 633. Eravacycline Use in Immunocompromised Patients: Multicenter Evaluation of Clinical and Safety Endpoints

**DOI:** 10.1093/ofid/ofac492.685

**Published:** 2022-12-15

**Authors:** Ashlan J Kunz Coyne, Sara Allosaimy, Kyle C Molina, Lena Kang-Birken, Abdalhamid M Lagnf, Kimberly C Claeys, Michael Veve, Madeline King, Benjamin Pullinger, Jeannette Bouchard, Tristan Gore, Leonar Rojas, Serina Tart, Athena L V Hobbs, Nicholas Perkins, Mark Biagi, Michael Pierce, Reese Cosimi, Bruce M Jones, James Truong, Justin Andrade, Glen Huang, Kristen Lucas, Taylor Morrisette, Dana Holger, Nicholas S Rebold, Amer El Ghali, Amer El Ghali, Susan L Davis, Michael J Rybak

**Affiliations:** Wayne State University, Detroit, Michigan; Wayne State, Detroit, Michigan; University of Colorado Hospital, Aurora, Colorado; Santa Barbara Cottage Hospital, Santa Barbara, California; Wayne State University, Detroit, Michigan; University of Maryland Baltimore, Baltimore, MD; Henry Ford Health System, Detroit, Michigan; Cooper University Hospital, Camden, New Jersey; University of the Sciences, Philadelphia, Pennsylvania; Prisma Health Richland - University of South Carolina, Columbia, South Carolina; University of South Carolina College of Pharmacy, Columbia, South Carolina; Valley Hospital Medical Center, Las Vegas, Nevada; Cape Fear Valley Health, Fayetteville, North Carolina; Cardinal Health Innovative Delivery Solutions, Dublin , Ohio; Baptist Memorial Hospital, Memphis, Tennessee; Swedish American Hospital, Rockford, Illinois; Swedish American Hospital, Rockford, Illinois; Ascension St Vincent, Indianapolis, Indiana; St. Joseph's/Candler Health System, Savannah, Georgia; Brooklyn Hospital, Brooklyn, New York; Brooklyn Hospital, Brooklyn, New York; University of California, Los Angeles, California; Wayne State University, Detroit, Michigan; Medical University of South Carolina College of Pharmacy, Charleston, South Carolina; Wayne State University, Detroit, Michigan; Wayne State University, Detroit, Michigan; Anti-infective Research Laboratory - Wayne State University, Detroit, Michigan; Anti-infective Research Laboratory - Wayne State University, Detroit, Michigan; Wayne State University, Detroit, Michigan; Eugene Applebaum College of Pharmacy and Health Sciences, Detroit, Michigan

## Abstract

**Background:**

Infections caused by multidrug-resistant (MDR) bacteria are an increasingly common public health threat associated with worse outcomes in immunocompromised patients. Eravacycline (ERV) has potent in-vitro activity against MDR Gram-negative and Gram-positive bacteria and has demonstrated non-inferiority to meropenem in the phase III IGNITE4 trial; however, the trial excluded immunocompromised patients. We aimed to evaluate clinical and safety endpoints of immunocompromised patients receiving ERV as definitive therapy.

**Methods:**

Multicenter, retrospective, observational study conducted from October 2018 to April 2022. Adult hospitalized immunocompromised patients treated with ERV for ≥72 hours were included. Immunocompromised patients were defined as having any of the following: chemo or radiation therapy < 30 days of hospital admission, HIV/AIDS with CD4 < 200, chronic steroids ( >40 mg prednisone or equivalent. The primary outcome was 30-day survival. Secondary outcomes were lack of 30-day infection recurrence and drug-related safety events.

**Results:**

Overall, 75 immunocompromised patients treated with ERV were included from 17 United States medical centers. Median (IQR) age was 62 (53-70) and 61.6% were male. Hospital length of stay was 28 (13-42) days and 67% were admitted to the intensive care unit. SOFA and APACHE II scores were 3.5 (1-7) and 16 (11-20), respectively. Common infection sources were intra-abdominal (26%) and lower respiratory tract (18%); 24% were bacteremic. Most patients had cultured Enterobacterales (58.7%) and Enterococci (37%) spp. infections. Of those, 21.3% were CRE and 19% were VRE. Infectious diseases consult was obtained in 91.8% of cases. Time elapsed from index culture collection to ERV initiation was 4 (2-8) days and duration of ERV therapy was 7 (4-12) days. In total, 81.3% of immunocompromised patients achieved 30-day survival and 90.7% did not have 30-day infection recurrence. Probable drug-related adverse events occurred in 5.3% of patients (GI 4%, rash 1%).

**Conclusion:**

A majority of immunocompromised patients receiving ERV as definitive therapy achieved 30-day survival and did not experience infection recurrence. ERV use in immunocompromised subpopulations will benefit from studies tailored to their specific characteristics.

**Disclosures:**

**Kimberly C. Claeys, PharmD**, BioFire Diagnostics: Honoraria **Bruce M. Jones, Pharm.D., FIDSA, BCPS**, AbbVie: Advisor/Consultant|AbbVie: Honoraria|La Jolla: Honoraria|Melinta: Advisor/Consultant|Paratek: Honoraria|Regeneron: Honoraria.

